# Incidental Discovery of a Phrygian Cap During Whipple's Procedure: A Case Report

**DOI:** 10.7759/cureus.59931

**Published:** 2024-05-08

**Authors:** Pratik S Navandhar, Pankaj Gharde, Raju K Shinde, Tushar Nagtode, Bhagyesh Sapkale, Varun Kulkarni

**Affiliations:** 1 General Surgery, Jawaharlal Nehru Medical College, Datta Meghe Institute of Higher Education and Research, Wardha, IND; 2 Medicine, Jawaharlal Nehru Medical College, Datta Meghe Institute of Higher Education and Research, Wardha, IND

**Keywords:** surgical intervention, gallbladder anomaly, case report, surgical management, incidental finding, cbd stenting, whipple's procedure, pancreatic cancer, obstructive jaundice, phrygian cap

## Abstract

Obstructive jaundice, characterised by yellow discolouration of the skin and mucous membranes due to reduced bile flow, often necessitates surgical intervention for resolution. This article provides a comprehensive literature review to contextualise the management of obstructive jaundice, focusing on common treatment modalities such as common bile duct (CBD) stenting and Whipple's procedure for pancreatic head cancer. Additionally, the incidental finding of a Phrygian cap of the gallbladder during surgical intervention for pancreatic head cancer is described in detail. A case presentation of a 48-year-old female with obstructive jaundice and pancreatic head cancer is outlined, detailing the diagnostic process, treatment decisions, and surgical interventions. The patient underwent CBD stenting followed by Whipple's procedure to address the pancreatic head cancer, during which the incidental discovery of a Phrygian cap of the gallbladder was noted. The discussion of the incidental finding highlights the complexity it adds to surgical interventions and emphasises the importance of adaptability and precision in managing anatomical variations. A comparison with similar cases underscores varying approaches to managing incidental findings, ranging from conservative observation to surgical excision based on clinical indications. This case underscores the significance of thorough diagnostic evaluation and surgical intervention in managing incidental findings such as the Phrygian cap, ensuring appropriate patient management and favourable clinical outcomes in complex surgical scenarios.

## Introduction

The yellow colouring of the skin and mucous membranes resulting from reduced bile flow is a frequent clinical disease known as obstructive jaundice [1]. Several conditions, such as strictures, cancers, or choledocholithiasis, can cause it [1,2]. The general goal of its management is to remove the obstruction, frequently through surgery or methods like common bile duct (CBD) stenting [2]. To keep the bile duct open and ensure unrestricted bile flow, a CBD stent is placed inside it [2,3]. A CBD stent can prevent further problems from arising and relieve symptoms like jaundice. The stent is usually implanted via an operation known as endoscopic retrograde cholangiopancreatography (ERCP), in which the bile ducts are accessed by passing a flexible tube equipped with a camera through the mouth, down the oesophagus, and into the stomach and small intestine [4].

The term "pancreatic head cancer" describes malignant growths seen in the pancreatic head, which is the portion of the pancreas next to the small intestine and bile duct [5]. A tumour marker called cancer antigen 19-9 (CA 19-9) may be present in higher amounts in the blood of certain pancreatic cancer patients [6]. A high CA 19-9 level in a patient with pancreatic head cancer indicates that the malignancy may be manufacturing this marker. Healthcare professionals can follow the progression of the illness and the patient's response to treatment by tracking CA 19-9 levels over time [5,6].

Pancreaticoduodenectomy, another name for Whipple's surgery, is a complex surgical treatment that is mainly used to address diseases affecting the duodenum, bile duct, and occasionally the stomach, as well as tumours and other abnormalities in the head of the pancreas [7]. Allen Whipple, the surgeon who invented the process in the 1930s, is honoured by the operation's name [7].

In radiology, a specific anatomical variation of the gallbladder is referred to as the Phrygian cap of the gallbladder [8]. It describes the rounded bottom portion of the gallbladder, the fundus, folding or infolding over the gallbladder body. The gallbladder resembles the recognisable Phrygian cap used in ancient Phrygian culture because of this folding, which gives it a slightly hood-like appearance [8]. Usually a benign anatomical variation, it might occasionally provide difficulties during gallbladder surgery or be linked to certain diseases like cholecystitis or gallstones [2,3]. A cholecystectomy, or surgical removal of the gallbladder, may be advised if the Phrygian cap is producing recurrent symptoms, complications such as gallstones or cholecystitis, or if there is a chance of issues in the future [4,8]. This can often be performed laparoscopically, which is less intrusive and heals more quickly than open surgery [3].

We aim to present a case study of a 48-year-old female with obstructive jaundice and pancreatic head cancer, detailing the diagnostic process, treatment decisions, and surgical interventions, with a focus on the management of the incidental Phrygian cap.

## Case presentation

We present the case of a 48-year-old female who presented to the Acharya Vinoba Bhave Rural Hospital (AVBRH) with complaints of jaundice and itching. Upon examination, she was diagnosed with obstructive jaundice. Further investigation revealed a small lesion on the head of the pancreas. Blood tests indicated elevated levels of cancer antigen 19-9 (CA 19-9), leading to a diagnosis of pancreatic head cancer.

Given the diagnosis, the patient underwent common bile duct (CBD) stenting to alleviate the obstructive jaundice. Blood tests and imaging examinations were performed, in addition to a comprehensive evaluation of the patient's medical history, to ascertain the type and extent of the obstruction before treatment. Prior to giving consent, the patient was informed about the procedure, its risks, benefits, and available options. The patient was prepped on the day of the procedure by fasting, setting up an intravenous (IV) line, and administering anaesthesia. A flexible endoscope was inserted through the mouth, oesophagus, stomach, and into the duodenum to start the stenting process. Imaging guidance was used to determine the location and extent of the obstruction in the bile duct. Dilation was used to open the constricted duct in preparation for the stent's implantation. Next, a tiny metal stent was placed over the blocked section of the bile duct using the endoscope. The correct placement of the stent was confirmed using imaging methods like endoscopy. After the surgery, the patient received post-procedure care instructions and was monitored for immediate issues.

Subsequently, a Whipple's procedure was planned to address the pancreatic head cancer. During the Whipple's procedure, or pancreaticoduodenectomy, an incidental finding of a Phrygian cap of the gallbladder was noted. The Whipple's procedure was meticulously carried out using standard protocol. The process began with the careful preparation of the patient, ensuring a sterile environment and the administration of general anaesthesia to induce unconsciousness and prevent pain throughout the surgery. Following this, a precise midline incision was made to access the abdomen, providing the necessary exposure for the subsequent steps.

The surgical team then embarked on an extensive exploration of the abdominal cavity, meticulously assessing the tumour's extent and identifying any additional abnormalities that could impact the procedure. With a clear understanding of the anatomy and pathology involved, the surgeon proceeded with careful dissection, isolating the head of the pancreas, duodenum, gallbladder, and common bile duct.

Subsequently, the affected portions of the pancreas, duodenum, and bile duct were meticulously excised, along with surrounding lymph nodes, ensuring the complete removal of cancerous tissue and minimising the risk of recurrence. The intricate reconstruction phase followed, wherein the remaining structures were connected to restore gastrointestinal continuity. This process involved careful pancreas, bile duct, and stomach reattachment, facilitating normal digestion and bile flow post-surgery.

**Figure 1:**
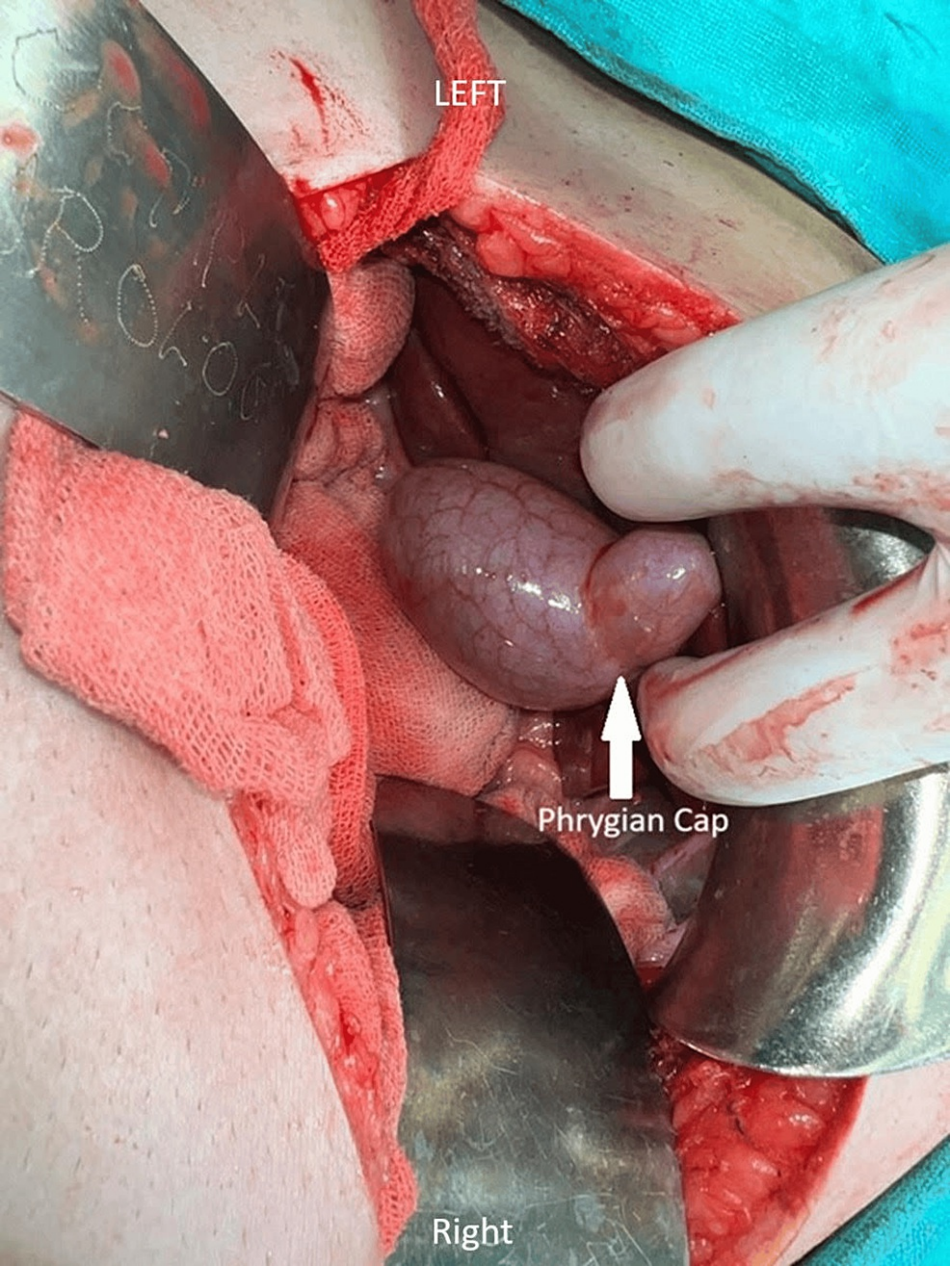
Phrygian cap of the gallbladder (white arrow)

**Figure 2:**
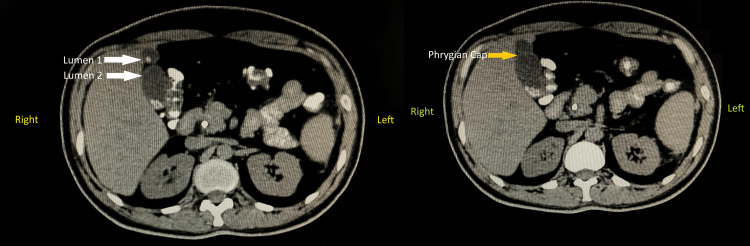
Computed tomography showing abnormal gallbladder and Phrygian cap Lumen 1 and Lumen 2 are highlighted by the white-coloured arrows, as the computed tomography image (left side) shows an abnormal gallbladder. The yellow-coloured arrow highlights the Phrygian cap in the given computed tomography image (right side).

Once the reconstruction was complete, the incisions were meticulously closed in layers to promote proper healing, and a drain was inserted to prevent fluid accumulation in the abdominal cavity, ensuring optimal postoperative recovery. Postoperative surveillance for dehiscence or fistula involved monitoring the surgical site and the patient's overall condition to detect any signs of complications, including visual inspection of the incision site for redness, swelling, or discharge, and gentle palpation for tenderness and abnormal tissue characteristics. Additionally, the patient was asked about any symptoms experienced, such as increased pain or fever. Throughout the procedure, utmost care and precision were exercised to minimise complications and maximise the patient's chances of a successful outcome. Additionally, the incidental finding of the Phrygian cap of the gallbladder was noted and managed appropriately within the context of the overall surgical plan. This anatomical variant was meticulously removed along with the primary focus of the surgery. The Phrygian cap of the gallbladder is shown in Figure 1. Computed tomography showing the abnormal gallbladder and Phrygian cap is represented in Figure 2.

Post-surgery, the patient experienced a recovery period of 20 days before being discharged from the hospital. Regular follow-up appointments were scheduled to monitor her progress and ensure optimal recovery. This case highlights the comprehensive approach taken in the management of complex conditions such as obstructive jaundice and pancreatic cancer. The incidental finding of the Phrygian cap underscores that close monitoring and follow-up care are essential for the long-term management and well-being of the patient. The timeline of patient care is described in Table 1.

**Table 1:** The timeline of patient care AVBRH: Acharya Vinoba Bhave Rural Hospital, CBD: common bile duct.

Event	Timeframe	Description
Initial presentation	Day 1	The patient arrives at AVBRH with jaundice and itching.
Diagnosis	Days 1-3	Examination reveals obstructive jaundice; imaging shows a pancreatic head lesion and blood tests indicate pancreatic cancer (CA 19-9).
Pre-treatment workup	Days 3-7	Blood tests, imaging, and medical history review are performed to assess the obstruction.
Informed consent	Day 5	The patient is informed about the CBD stenting procedure, risks, benefits, and alternatives.
CBD stenting procedure	Day 7	The patient undergoes fasting, IV placement, and anaesthesia. A flexible endoscope is used to locate the obstruction, widen the bile duct, and insert a stent. Stent placement is confirmed with imaging.
Post-stenting care	Days 7-10	The patient receives instructions and is monitored for post-procedure issues.
Planning for Whipple's procedure	Days 10-14	The decision is made to perform surgery for pancreatic cancer. Additional tests may be scheduled.
Whipple's procedure	Day 14	The patient's tumour and the incidental Phrygian cap of the gallbladder are removed along with the reconstruction of the pancreas and surrounding structure.
post-operative recovery	Days 14-34	The patient recovers in the hospital for 20 days, with additional recovery time at home likely needed.
Discharge and follow-up	Day 34	The patient is discharged with follow-up appointments scheduled.

## Discussion

The incidental discovery of a Phrygian cap of the gallbladder during a Whipple's procedure for pancreatic head cancer in a 48-year-old female presents a unique and noteworthy finding. The Phrygian cap anomaly, characterised by a fold or hooding of the gallbladder fundus over the body, is a rare congenital variation often detected incidentally during imaging or surgical procedures [8]. In this case, the discovery of the Phrygian cap adds a layer of complexity to an already intricate surgical intervention for pancreatic head cancer. The presence of a Phrygian cap may alter the usual anatomical landmarks, requiring adaptability and precision to avoid accidental injury to the gallbladder or bile ducts [5].

In the case of a 65-year-old female reported by Kulkarni et al., the Phrygian cap of the gallbladder was discovered during an emergency exploratory laparotomy undertaken for a sealed-off perforation at the pyloric part of the stomach [9]. In our case of a 48-year-old female, it was incidentally noted during a Whipple's procedure performed for pancreatic head cancer. Both cases involve the identification of a gallbladder anomaly during surgical intervention for other conditions. However, the approach to managing the gallbladder anomaly differs significantly between the two cases [9]. In our case, where the patient underwent a Whipple's procedure, the Phrygian cap was meticulously removed along with the primary focus of the surgery, namely pancreatic head cancer. This approach aligns with the principle of comprehensive surgical intervention aimed at addressing all identifiable pathologies within the operative field.

Conversely, in the case reported by Kulkarni et al., the anomaly was asymptomatic, and the operating surgeons opted against further dissection, likely due to the absence of clinical indications necessitating intervention [9]. Instead, the surgery addressed the primary pathology, namely the sealed-off perforation at the pyloric part of the stomach, through Graham's patch repair. This decision reflects a more conservative approach, prioritising the management of symptomatic conditions while avoiding unnecessary interventions for asymptomatic anomalies.

In the case of a 41-year-old female patient reported by Ogut et al., presenting with symptoms of acute cholecystitis, including severe upper right abdominal pain, nausea, vomiting, and fever [10], despite a history of peptic ulcer disease, diagnostic imaging revealed gallbladder duplication with stones. Emergency open cholecystectomy was performed due to the complexity of the case, revealing two gallbladders with corresponding cystic ducts and an anomaly involving the right hepatic duct [10]. Successful removal of both gallbladders was achieved, ensuring patient safety and recovery. Follow-up confirmed a favourable outcome. This case underscores the significance of thorough exploration and open surgery in managing anatomical variations like gallbladder duplication, promoting optimal patient outcomes [10].

The incidental finding of a Phrygian cap of the gallbladder in the case of the 81-year-old male undergoing right hemicolectomy and liver resection for adenocarcinoma with liver metastasis presents an interesting comparison to a similar finding in a younger patient undergoing Whipple's procedure or pancreaticoduodenectomy, as reported in this case of a 48-year-old female [11]. In both cases, the Phrygian cap anomaly of the gallbladder was noted incidentally during surgical procedures aimed at addressing primary malignancies in other organs. The advanced age of the patient, in the case of the 81-year-old male reported by van Kamp et al., adds complexity due to the presence of multiple comorbidities and the need for simultaneous surgical interventions for colon cancer and liver metastasis [11]. The decision to perform cholecystectomy to facilitate liver resection highlights the importance of thorough perioperative evaluation and strategic planning to optimise surgical outcomes, especially in elderly patients with significant medical history. Both cases highlight the importance of recognising and addressing incidental anatomical variations, such as the Phrygian cap, during surgical interventions to optimise patient care and surgical outcomes.

In the case of a 53-year-old female reported by Al-Ashqar et al., she initially presented with a three-month history of right upper quadrant abdominal pain radiating to her back, accompanied by nausea and unintentional weight loss [12]. Diagnostic laparoscopy confirmed the presence of a folded fibrous cap on the gallbladder fundus, consistent with a Phrygian cap, alongside microscopic evidence of chronic cholecystitis. The patient underwent laparoscopic cholecystectomy, leading to complete resolution of symptoms. In our case of the 48-year-old female during a Whipple's procedure, incidental Phrygian cap discovery was observed. In contrast, in the 53-year-old female case reported by Al-Ashqar et al., laparoscopic cholecystectomy following diagnostic laparoscopy confirmed the presence of a Phrygian cap [12]. Both these cases show the importance of proper diagnosis and surgery in managing incidental findings, which helps manage the patient's condition and treatment properly.

## Conclusions

The case report details the management of obstructive jaundice and pancreatic head cancer in a 48-year-old female, complicated by the incidental discovery of a Phrygian cap of the gallbladder during Whipple's procedure. The treatment involved CBD stenting for jaundice and Whipple's surgery for cancer, with meticulous attention to the incidental findings. Surgical adaptability and careful decision-making were crucial, highlighting the importance of addressing primary and incidental pathologies for optimal patient outcomes. Postoperative monitoring and patient-centred care were emphasised. The case underscores the complexity of surgical interventions and the significance of thorough management in achieving favourable clinical results.

## References

[1] Wani BN, Jajoo SN (2009). Obstructive jaundice in neonates. Trop Gastroenterol.

[2] Modha K (2015). Clinical approach to patients with obstructive jaundice. Tech Vasc Interv Radiol.

[3] Barai V, Hedawoo J, Changole S (2017). Forgotten CBD stent (102 months) with stone-stent complex: a case report. Int J Surg Case Rep.

[4] Syrén EL, Sandblom G, Enochsson L, Eklund A, Isaksson B, Österberg J, Eriksson S (2022). Outcome of ERCP related to case-volume. Surg Endosc.

[5] Olakowski M, Grudzińska E (2023). Pancreatic head cancer - current surgery techniques. Asian J Surg.

[6] Jia YH, Zheng WW, Ye ZH (2020). Clinical significance of CA 19.9 and LINC01197 in pancreatic cancer. Eur Rev Med Pharmacol Sci.

[7] Guerrero-Martínez GA, Estrada-Gómez R, Basilio-Roque A, Viveros-Luna R, Lorenzo-Yacamix C, Dávila-Esparza PA (2020). Laparoscopic pancreatoduodenectoy. Cir Cir.

[8] Kokilavani J, Indiran V (2018). Phrygian cap. Abdom Radiol (NY).

[9] Kulkarni V, Ramteke H, Lamture Y, Gharde P, Nagtode T, Rewale V (2022). A rare incidental finding of Phrygian cap in a case of pyloric perforation. Cureus.

[10] Ogut E, Yildirim FB, Memis O (2024). Duplicated gallbladder with acute cholecystitis: a case of unusual presentation and diagnostic challenges. World J Emerg Med.

[11] van Kamp MJ, Bouman DE, Steenvoorde P, Klaase JM (2013). A phrygian cap. Case Rep Gastroenterol.

[12] Al-Ashqar M, Maliyakkal AK, Shiwani MH, Anwar S (2013). Acalculous Phrygian cap cholecystitis. BMJ Case Rep.

